# Delayed development of systemic immunity in preterm pigs as a model for preterm infants

**DOI:** 10.1038/srep36816

**Published:** 2016-11-10

**Authors:** Duc Ninh Nguyen, Pingping Jiang, Hanne Frøkiær, Peter M. H. Heegaard, Thomas Thymann, Per T. Sangild

**Affiliations:** 1 Section of Comparative Pediatrics and Nutrition, Department of Veterinary Clinical and Animal Sciences, University of Copenhagen, Denmark; 2 Department of Veterinary Disease Biology, University of Copenhagen, Denmark; 3 National Veterinary Institute, Technical University of Denmark, Frederiksberg, Denmark; 4 Department of Pediatrics and Adolescent Medicine, Rigshospitalet, Copenhagen, Denmark

## Abstract

Preterm neonates are highly sensitive to systemic infections in early life but little is known about systemic immune development following preterm birth. We hypothesized that preterm neonates have immature systemic immunity with distinct developmental trajectory for the first several weeks of life, relative to those born at near-term or term. Using pigs as a model, we characterized blood leukocyte subsets, antimicrobial activities and TLR-mediated cytokine production during the first weeks after preterm birth. Relative to near-term and term pigs, newborn preterm pigs had low blood leukocyte counts, poor neutrophil phagocytic rate, and limited cytokine responses to TLR1/2/5/7/9 and NOD1/2 agonists. The preterm systemic responses remained immature during the first postnatal week, but thereafter showed increased blood leukocyte numbers, NK cell proportion, neutrophil phagocytic rate and TLR2-mediated IL-6 and TNF-α production. These immune parameters remained different between preterm and near-term pigs at 2–3 weeks, even when adjusted for post-conceptional age. Our data suggest that systemic immunity follows a distinct developmental trajectory following preterm birth that may be influenced by postnatal age, complications of prematurity and environmental factors. Consequently, the immediate postnatal period may represent a window of opportunity to improve innate immunity in preterm neonates by medical, antimicrobial or dietary interventions.

Every year, 15 million infants are born preterm (<37 weeks of gestation) with a mortality rate of up to 10%^1^. Those who are born very preterm (<32 weeks) are extremely susceptible to infections and intestinal diseases due to the immaturity of the gastrointestinal tract and immune system, i.e. 20–40% of these hospitalized infants develop necrotising enterocolitis (NEC) and/or sepsis^1^,^2^,^3^,^4^. NEC affects 7% of very low birth weight infants (<1500 g, VLBW) with a mortality of up to 50% for those subjected to surgical intervention^5^. The pathogenesis and etiology of NEC are still incompletely understood after several decades of research but prematurity, abnormal gut bacterial colonization and aggressive enteral feeding, especially with infant formula, may be key predisposing factors^5^. For sepsis, mortality is much lower than for NEC, but the incidence is much higher, up to 60% in VLBW infants^6^. Early onset sepsis (EOS) occurs within the first 3 days after birth and is thought to be caused mainly by maternal transfer of invasive microorganisms^7^. Late-onset sepsis (LOS) is defined as sepsis after day 3 and is more likely derived from postnatal acquisition of pathogens from the environment, including *S. epidermidis*, partly entering the body via indwelling catheters^7^,^8^. In addition, LOS may result from bacterial translocation across the immature gut, as blood cultures from neonates with LOS and bacteremia often reflects their gut bacterial composition^9^. Infection induced by bacterial translocation may be most prevalent shortly after preterm birth and before gut closure, especially when the immature gut has not yet been exposed to the maturational and barrier-supporting effects of mother’s milk (e.g. sIgA for passive immunity)^10^. Both LOS and NEC can progress rapidly after clinical presentation, and accurate diagnosis is important to initiate proper therapy such as antibiotics, bowel rest or gut surgery in case of severe NEC. Despite tremendous efforts to identify diagnostic markers for LOS and NEC (e.g. C-reactive protein, IL-6, serum amyloid A), none of them are accurate and specific enough for clinical use^11^.

Birth represents a sudden change from a protected intrauterine environment to the extrauterine environment with exposure to billions of bacteria^6^. An immune-suppressed physiological state is required for the fetus^8^, but this must change after birth to cope with environmental bacteria. In newborn preterm infants, blood leukocytes are deficient in expression of surface receptors (CD14, MD-2, Toll-like receptors, TLRs)^12^, leading to impaired immune response upon bacterial challenges and higher susceptibility to infectious diseases^8^. For example, deficient TNF-α and IFN-γ responses impair Th1 polarization and neutrophil functions^13^. Preterm infants also have lower levels of antimicrobial factors in various organs (e.g. bactericidal/permeability-increasing protein, defensins and cathelicidin in the lungs, gut and circulation)^14^, poor phagocytic rate of blood neutrophils and monocytes^15^, and reduced capacity to release neutrophil extracellular traps (NETs) for bacterial killing^16^.

We have established a model in preterm pigs that displays many of the clinical signs present after preterm birth in infants (e.g. immature function of many organ systems, including lungs, together with high sensitivity to NEC and LOS) when piglets are born at 90% gestation^9^,^17^,^18^. Direct comparison of 90% gestation preterm pigs with preterm infants is difficult and depends on gestational age (e.g. 60–80% in preterm infants) and varies among organs (e.g. lungs, gut, liver, brain). Regardless, the model allows study of multiple clinically-relevant treatments, such as respiratory and cardiovascular support, together with parenteral and enteral nutrition with repeated blood sampling for characterization of immune parameters. Cord blood of preterm pigs has very low leukocyte numbers, especially neutrophil numbers, compared with term and adult pigs^9^, which is similar to conditions in newborn preterm infants^12^. Blood neutrophils are the major innate cell subset and key effector of the innate immune defense with capacity of exerting antimicrobial functions such as phagocytosis, NETs and modulation of cytokine response^15^,^16^,^19^,^20^. Newborn preterm pigs are severely neutropenic and neutrophil counts do not change significantly during the first five days of life, probably in part explaining the poor innate host defense during this period^9^. In both preterm infants and pigs, LOS and NEC occur most frequently during the first few weeks of life but it remains unclear if this is mirrored by impaired systemic immunity at this time^7^,^17^,^21^,^22^. It is important to characterize the nature of impaired immunity in preterm neonates to identify critical periods of development and factors that may reduce the susceptibility to infections.

We hypothesized that newborn preterm pigs would have an immature innate immunity with distinct developmental trajectory, relative to pigs born close to term, during the first weeks of postnatal life when infection susceptibility is high. We analyzed a series of innate immune parameters of blood leukocytes at birth and during the first four weeks of life in preterm pigs, relative to pigs born near-term or at term. Our results will help to clarify how the immature systemic immune system in preterm neonates adapts after birth and how this adaptation may be influenced by various factors in the postnatal period.

## Results

### Clinical evaluation

A total of 114 preterm pigs (90% of gestation) from 5 sows and 21 near-term pigs (98% of gesation) from one sow were delivered with birth weights of 500–1400 g. Twenty-two preterm pigs died shortly after delivery due to typical signs of prematurity (respiratory distress) and were excluded from the study. Ninety-two preterm and 21 near-term pigs were included in analyses. Six preterm and seven near-term pigs were euthanized before the planned end of the study (weeks 3–4) due to variable clinical signs, including dehydration, diarrhea and body weight loss.

**Figure 1:**
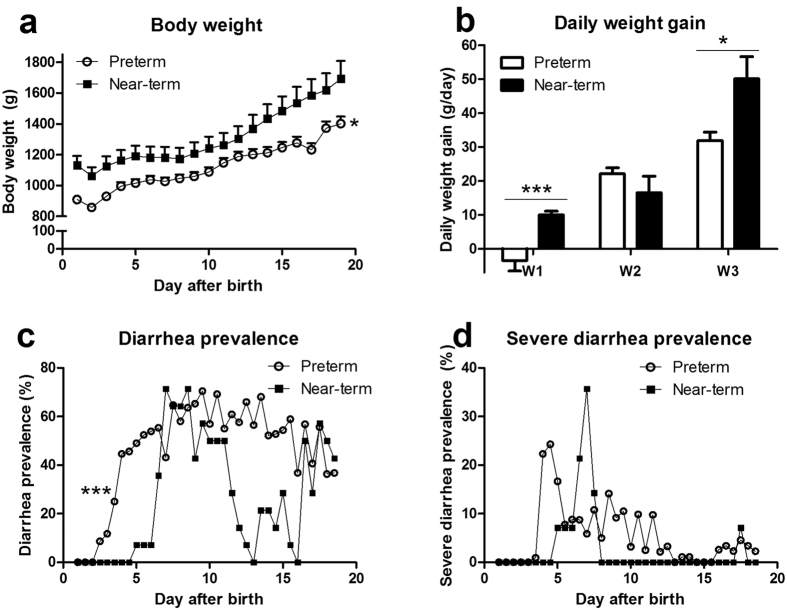
Postnatal growth and diarrhea in preterm and near-term pigs. (**a**) body weight; (**b**) daily weight gain; (**c**) diarrhea incidence (fecal score >2). (**d**) severe diarrhea incidence (fecal score >4). A total of 92 preterm and 14 near-term pigs were included in the analysis. * and ***P < 0.05 and 0.001, respectively, between preterm and near-term pigs.

During the first four weeks of life, both preterm and near-term pigs were fed a NEC-protective diet of raw bovine milk and no pigs showed any clear signs of NEC, sepsis or organ failure by the end of the experiment. From week two, preterm pigs commonly showed poor weight gain and diarrhea. Preterm pigs had lower body weight across the study period, and lower daily weight gain during week 1 and 3, relative to near-term pigs (P < 0.05, Fig. 1a,b). Diarrhea prevalence was higher in preterm vs. near-term pigs (P < 0.001, Fig. 1c). Less than 10% of near-term pigs had severe diarrhea across the study (except days 6–7 with 20–35%) whereas 10–25% preterm pigs had severe diarrhea during days 4–12 (Fig. 1d).

### Preterm cord blood has low leukocyte counts and impaired responses to inflammatory challenges

**Figure 2:**
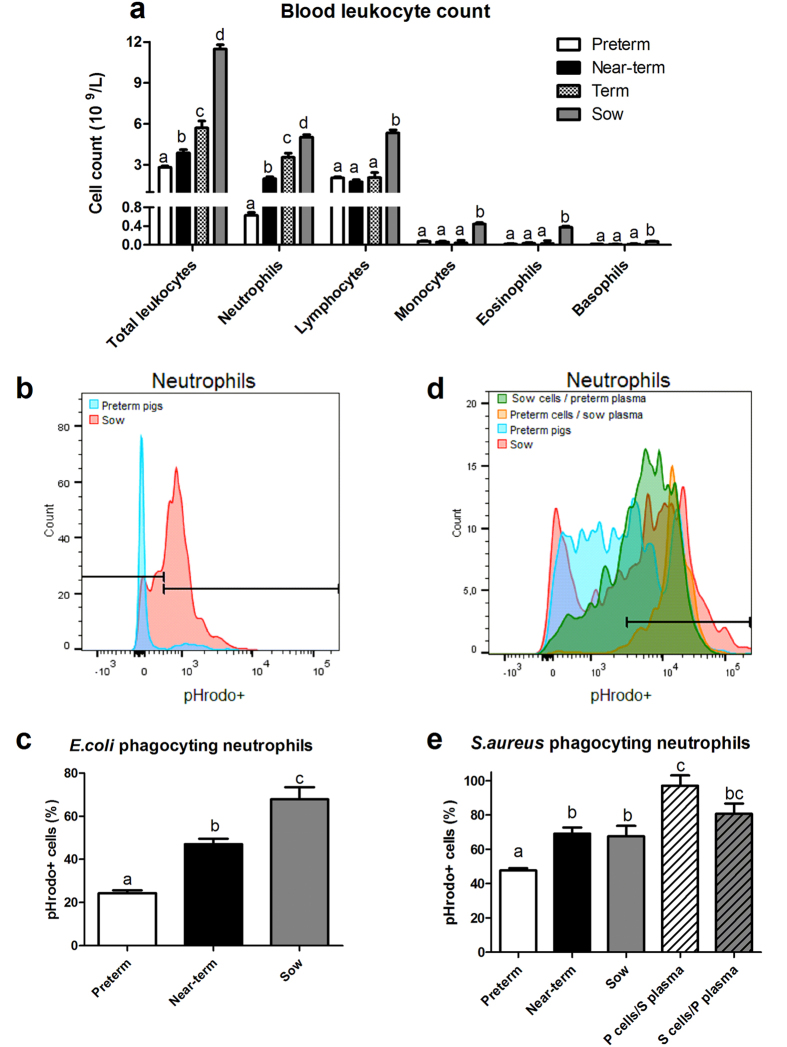
Blood leukocyte counts and phagocytosis activity in cord blood and venous sow blood. (**a**) Blood leukocyte counts in cord blood of preterm (n = 92), near-term (n = 21) and term (n = 4) pigs and venous sow blood (n = 11). Blood neutrophil phagocytic rate, demonstrated as proportions of neutrophils exerting phagocytosis, against *E. coli* (**b,c**) and *S. aureus* (**d,e**) in newborn preterm pigs (n = 60–67), newborn near-term pigs (n = 9–21), their mother sow (n = 3–4), or from preterm blood cells with sow plasma (P cells/S plasma, n = 3), or sow blood cells with preterm plasma (S cells/P plasma, n = 3). (**b,d**) Representative histogram showing higher proportions of pHrodo+ neutrophils (the gate on the right) following blood challenge with *E. coli* and *S. aureus*. Values in the same cell population not sharing similar letters are significantly different (P < 0.01).

We demonstrated different degrees of immature systemic immunity in preterm pigs at birth by comparing leukocyte counts, neutrophil phagocytosis against both Gram-positive and –negative bacteria and ability of *in vitro* NET formation in the cord blood of preterm, near-term and term pigs and venous sow blood (Fig. 2). Total blood leukocyte and neutrophil counts followed similar trends: preterm < near-term < term < sow (P < 0.01, Fig. 2a). Blood lymphocyte, monocyte, eosinophil and basophil counts among preterm, near-term and term pigs were similar, but all these values were lower than in sows (P < 0.01, Fig. 2a).

Neutrophil phagocytic rate (proportion of neutrophils exerting phagocytosis) of *E. coli* was lowest in preterm cord blood (~20%), followed by near-term cord blood (~40%) and highest in adult blood (~70%, P < 0.05, Fig. 2b,c). Near-term cord blood and adult blood had similar percentage of neutrophils phagocytosing *S. aureus*, but the values were higher than in preterm cord blood (~70% vs. 50%, P < 0.01, Fig. 2d–e). Of note, artificial samples, consisting of isolated preterm cord blood cells combined with plasma extracted from sow blood, had markedly elevated number of phagocytic neutrophils than both preterm and sow blood (P < 0.001, Fig. 2e), indicating the important role of plasma soluble mediators in bacterial killing. In contrast, these phagocytic parameters from samples consisting of isolated sow blood cells combined with preterm plasma were similar to that from sow blood.

The ability of *in vitro* NET formation were quantified by the released amount of cell-free DNA (cfDNA) in the blood after stimulation with phorbol 12-myristate 13-acetate (PMA) or lipopolisaccharide (LPS). Neutrophils from cord blood of preterm pigs did not have any ability to form NETs *in vitro* (Supplementary Fig. S1a). Systemic injection of *S. epidermidis* into preterm pigs immediately after birth did not elevate plasma cfDNA levels either, indicating no *in vivo* NET formation under bacterial challenge (Supplementary Fig. S1b).

### Preterm cord blood has impaired TLR-mediated cytokine production

**Figure 3:**
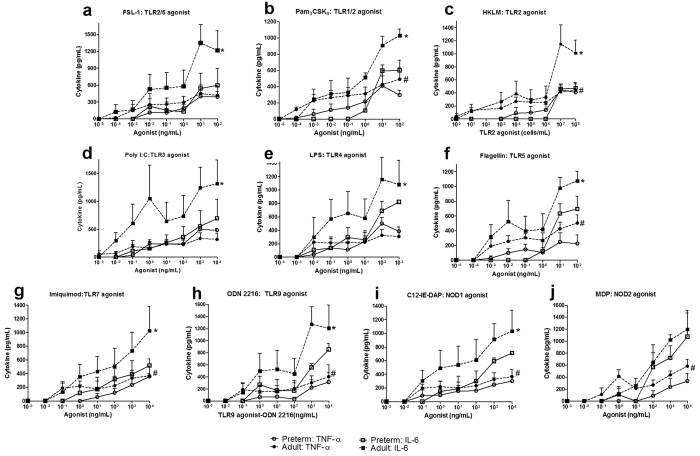
Pattern recognition receptor-mediated TNF-α and IL-6 production in the blood of preterm pigs at birth and adult pigs. For each litter (n = 3), pooled whole blood of all preterm pigs at birth and blood of the sow were stimulated with 10 TLR and NOD agonists for five hours prior to plasma collection for cytokine analysis. (**a**) TLR2/6. (**b**) TLR1/2. (**c**) TLR2. (**d**) TLR3. (**e**) TLR4. (**f**) TLR5. (**g**) TLR7. (**h**) TLR9. (**i**) NOD1. (**j**) NOD2. ^*,#^P < 0.05 for comparisons of IL-6 and TNF- α between preterm and adult pigs.

The systemic immune responses in preterm pigs at birth were quantified by inflammatory cytokine production following *in vitro* stimulation of whole blood with multiple agonists of TLRs or NOD-like receptors. Among 10 different agonists, preterm cord blood had lower TNF-α and IL-6 production in response to TLR1/2, TLR2, TLR5, TLR7, TLR9 and NOD1 agonists, compared with adult blood (P < 0.05, Fig. 3b,c,f–i). TLR2/6, TLR3, and TLR4-induced TNF-α levels were similar between preterm cord and adult blood (Fig. 3a,d,e). NOD2-induced IL-6 levels were also similar between preterm cord and adult blood (Fig. 3j). In particular, preterm cord blood had no response to TLR2 and TLR5 to induce IL-6, and a limited TNF-α response to TLR7 and NOD2 agonists at most of the tested concentrations.

### Postnatal development of blood cell subsets and plasma mediators

**Figure 4:**
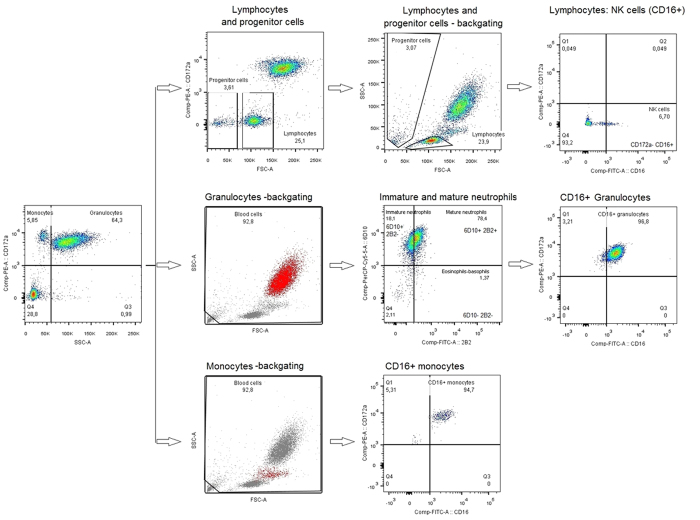
Gating strategy. Monocytes (CD172a^++^ SSC^low^) and granulocytes (CD172a^+^ SSC^high^) were gated based on granularity and CD172a, then confirmed by their locations in the original FSC/SSC plot. Granulocytes were further sub-divided into immature neutrophils (CD172a^+^ SSC^high^ 6D10^+^ 2B2^-^) and mature neutrophils (CD172a^+^ SSC^high^ 6D10^+^ 2B2^+^). Lymphocytes were gated based on CD172a and its location in FSC/SSC plots (CD172a^−^ FSC^medium^). NK cells were defined as CD16^+^ lymphocytes.

Various subsets of blood leukocytes including immature and mature neutrophils, NK cells and CD16^+^ monocytes during the first four weeks of life in preterm pigs were characterized by flow cytometry (see Methods). Gating strategy of cell subset was demonstrated in Fig. 4.

**Figure 5:**
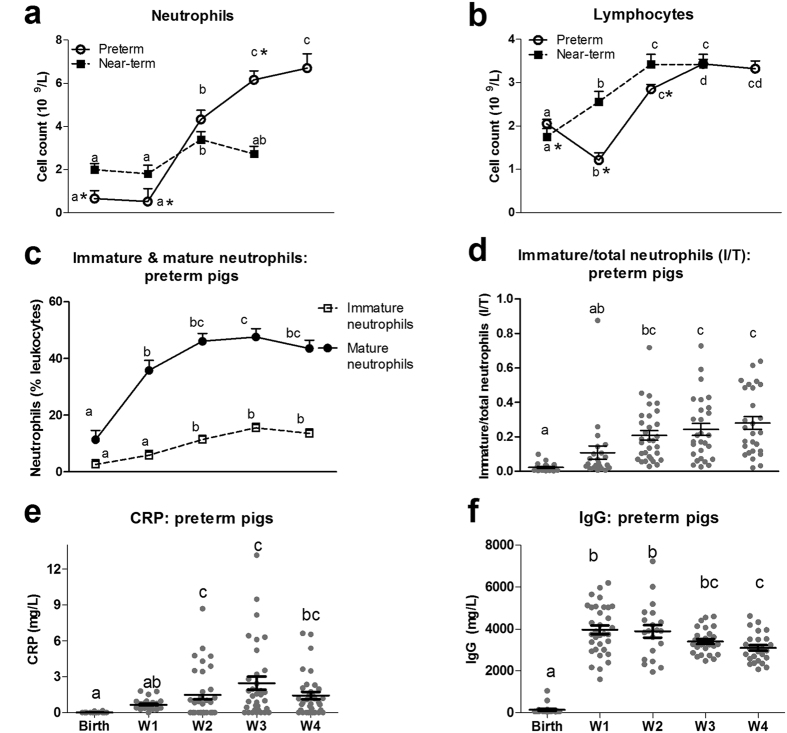
Development of blood leukocyte subsets and plasma components during the first four weeks of life in preterm and near-term pigs. (**a**) Absolute neutrophil counts. (**b**) Absolute lymphocyte counts. (**c**) Frequency of immature (CD172a^+^ SSC^high^ 6D10^+^ 2B2^-^) and mature neutrophils (CD172a^+^ SSC^high^ 6D10^+^ 2B2^+^) in total blood leukocytes. (**d**) The ratio of immature neutrophils in total number of neutrophils (I/T ratio). (**e,f**) Plasma CRP and IgG levels. A sub-population of preterm (n = 34–92) and term pigs (n = 12–21) was analyzed. Values of the same parameter during the four weeks not sharing similar letters are significantly different (P < 0.01 for **a,b,** P < 0.05 for **c–f**). *P < 0.05, compared between preterm vs. near-term pigs at the same time period.

Absolute neutrophil and lymphocyte counts of preterm pigs were constant during the first week of life and increased from week 2 (8-fold for neutrophils, 1.5-fold for lymphocytes, P < 0.01, Fig. 5a,b). In near-term pigs, blood neutrophil and lymphocyte counts were higher than in preterm pigs during the first week of life (P < 0.05) but they also increased in numbers in week 2 (P < 0.05, Fig. 5a,b). Preterm and near-term pigs showed similar neutrophil and lymphocyte counts in week 2. Importantly in week 3, blood neutrophil counts in preterm pigs reached higher levels than in near-term pigs (P < 0.05, Fig. 5a). When comparing the groups at the same post-conceptional age, preterm pigs in week 2 (day 10–11) had higher blood neutrophil and lymphocyte counts than near-term pigs at birth (born 10 days later than preterm pigs, P < 0.05).

Preterm pigs were further characterized with parameters related to systemic inflammation. The amount of mature neutrophils increased rapidly after preterm birth and reached a constant proportion of about 40% of blood leukocytes during weeks 1–4, while immature neutrophil frequency gradually increased from 2–5% during week 1 to 10% of blood leukocytes during weeks 2–4 (P < 0.05, Fig. 5c). Correspondingly, the ratio of immature to total number of neutrophils (I/T ratio) was relatively low at preterm birth and week 1 (~0.1), but increased in weeks 2–4 (~0.2, P < 0.05, Fig. 5d). It was noteworthy that male pigs had greater frequency of immature neutrophils than female pigs (P < 0.05). Plasma CRP in preterm pigs was undetectable at preterm birth, increased marginally in week 1, but more markedly in weeks 2–4 (to ~3000 μg/L, P < 0.05, Fig. 5e). All pigs had plasma CRP levels <10 mg/L (levels for suspected infection in preterm infants), except 2 pigs during week 2 and 1 pig during weeks 3–4. However, these 3 pigs did not have any other signs of infection and had normal CRP levels in the following measurements. Plasma IgG levels were also very low at preterm birth but increased to a stable level of ~4000 mg/L in weeks 1–4, in response to infusion of sow’s plasma on day 1 (P < 0.05, Fig. 5f).

### Postnatal development of *in vitro* neutrophil phagocytosis and NET formation in the blood

**Figure 6:**
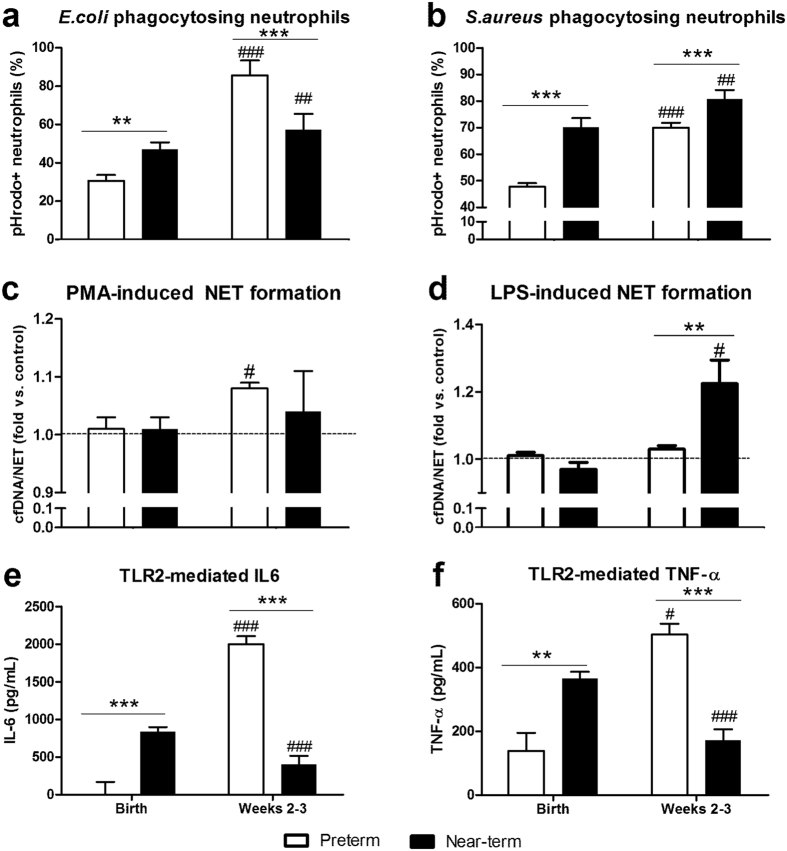
Development of systemic immune responses during the first three weeks of life in preterm and near-term pigs. (**a,b**) Proportion of neutrophils exerting phagocytosis in the blood against *E. coli* (n = 31–32 for preterm, n = 14–21 for near-term) and *S. aureus* (n = 55–60 for preterm, n = 9–15 for near-term). (**c,d**) NET response *in vitro*, shown by the relative increase of plasma cfDNA in samples with stimulation vs. control, following blood stimulation with PMA 100 ng/mL or LPS 1 μg/mL for 3 h (n = 51–58 for preterm, n = 12–20 for near-term pigs). (**e,f**) TLR2-mediated production of IL-6 and TNF-α in whole blood (n = 12 for preterm, n = 12–21 for near-term). In (**a,b,e,f**) ^#, ##, ###^P < 0.05, 0.01 and 0.001, compared with corresponding values at birth. In (**c,d**) ^#^P < 0.05, compared with the corresponding control without stimulation, which was assigned with an arbitrary unit of 1. ** and ***P < 0.01 and 0.001, compared between preterm vs. near-term pigs at the same time period.

The phagocytic rates of blood neutrophils towards *E. coli* and *S. aureus* increased after birth but at very different rates in preterm and near-term pigs (P < 0.01, Fig. 6a,b). Preterm blood neutrophil phagocytic rate was low at birth but increased dramatically in weeks 2–3, e.g. 2.7-fold for *E. coli* and 1.7-fold for *S. aureus* (P < 0.001, Fig. 6a,b). Relative to preterm pigs, blood neutrophil phagocytic rates in near-term pigs at birth were higher, and increased more moderately over time (1.2-fold, for both *E. coli* and *S. aureus*, P < 0.01). In weeks 2–3, preterm pigs had lower frequency of *S. aureus* phagocytosing neutrophils, but higher frequency of *E. coli* phagocytosing neutrophils, than near-term pigs (P < 0.001, Fig. 6a,b). At the same post-conceptional age, preterm pigs in week 2 had higher frequency of *E. coli* phagocytosing neutrophils than near-term pigs at birth (P < 0.05).

From negligible *in vitro* NET response at birth, both preterm and near-term pigs had increased NET response in weeks 2–3, as shown by elevated plasma cfDNA release following PMA stimulation in preterm pigs and following LPS stimulation in near-term pigs (P < 0.05, Fig. 6c,d). Specifically, LPS-induced cfDNA release was higher in near-term than in preterm pigs in weeks 2–3 (P < 0.001, Fig. 6d).

### Postnatal development of TLR2-mediated cytokine production

Because a large proportion of preterm neonates are susceptible to Gram-positive bacterial infection during early life, we chose to characterize the development of systemic cytokine response in preterm pig blood challenged with TLR2 agonist. Preterm and near-term pigs showed differential development of TLR2-mediated IL-6 and TNF-α production in whole blood. TLR2-mediated cytokine responses in preterm pigs were negligible at birth but increased markedly in weeks 2–3 (P < 0.001, Fig. 6e,f), potentially indicating immune maturation. In contrast, the IL-6 and TNF-α responses in near-term pigs were higher at birth (P < 0.01), but decreased slightly by weeks 2–3 (P < 0.001, relative to at birth) to levels that were lower than in preterm pigs (P < 0.001, Fig. 6e,f). All cytokines values in preterm pigs in week 2 were higher than in near-term pigs at birth (e.g. comparison at the same post-conceptional age, P < 0.05).

### Postnatal development of NK cells and CD16^+^ monocytes

**Figure 7:**
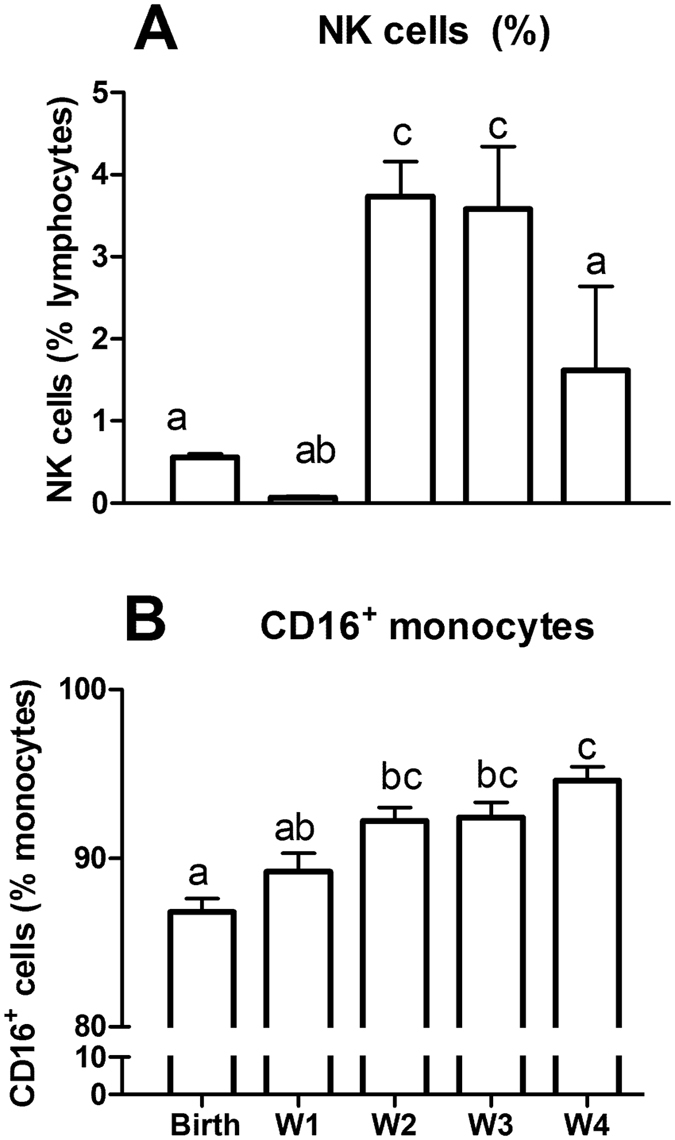
The development of NK cells (CD16^+^ lymphocytes) and CD16^+^ monocytes during the first four weeks of life in preterm pigs. **(A**) Frequency of NK cells in lymphocytes. (**B**) Frequency of CD16+ monocytes in total monocyte number (n = 21–33). Values not sharing similar letters are significantly different (P < 0.05).

We also evaluated the frequency of NK cells, indicating the capacity to eliminate virally-infected cells^23^ and CD16^+^ monocytes (monocytes with expression of Fcγ receptor) that recognize Ig, an opsonin to facilitate phagocytosis^24^. Within the first week, preterm pigs had less than 1% of NK cells in the lymphocyte population but this proportion increased to 3.5% in weeks 2–3 and the decreased slightly in week 4 (P < 0.05, Fig. 7A). CD16^+^ monocyte frequency increased gradually and reached 92–95% in weeks 2–4, compared with 86–90% at birth and week 1 (P < 0.05, Fig. 7B).

## Discussion

We have described the development of the systemic innate immunity during the first few weeks after preterm birth in pigs, aiming to better understand the high susceptibility to systemic infections in preterm infants. At birth and during the first week of life, preterm pigs showed very low blood neutrophil and lymphocyte counts, negligible NK cell frequency, low TLR-mediated cytokine production and impaired phagocytosis and NET responses. This may relate to the previously observed high incidence of bacteremia, osteomyelitis and NEC in formula-fed preterm pigs^9^,^25^,^26^,^27^. We identified a critical period during the second week of life where many innate immune parameters started to mature (blood neutrophil counts, NK cells and CD16^+^ monocyte frequencies, TLR-mediated cytokine production, phagocytosis and NET formation). This, together with the provision of enteral antibiotics when indicated, may have helped to secure that our milk-fed preterm pigs showed no signs of systemic or gut infections. Nevertheless, the NET response and neutrophil phagocytic rate of Gram-positive bacteria remained less potent in preterm vs. near-term pigs in weeks 2–3, suggesting a persistent delay for some innate immune functions. Conversely, TLR2-induced cytokine responses and neutrophil phagocytosis of Gram-negative bacteria in weeks 2–3 were higher in preterm vs. near-term pigs, indicating a high degree of postnatal compensatory response to preterm birth. Overall, our study suggests that systemic immunity in preterm pigs is immature during the first week of life and develop and mature in a distinct postnatal trajectory, relative to that in near-term pigs at the same postnatal age. Of note, when the immune parameters were compared at the same post-conceptional age, e.g. between preterm pigs at day 10–11 and near-term pigs at birth (delivered 10 days later than preterm), all measured parameters differed. This suggests that the developmental trajectory of systemic immunity in preterm neonates reflects more than just a response to advancing ontogenic age, but is affected also by factors such as external environment, variable antibiotic treatment and the body responses to preterm birth (e.g. impaired organ functions including a degree of respiratory distress).

For comparison between infants and pigs in this context, it could be important that preterm infants are born with reduced immunoglobulin (Ig) levels following their shortened time for transplacental transport of Igs before birth^28^, while both preterm and near-term pigs are devoid of any IgG passage from their mothers before birth^29^,^30^. In pigs, this passive immunity is normally transferred to the newborns via their mother’s colostrum in the days just after birth, making use of the endocytic capacity of pig intestinal enterocytes to absorb Igs. This Ig uptake capacity is also present in the intestine of preterm pigs although lower than in near-term pigs^31^. Both IgG and factors such as CRP are known to be important mediators of immune functions and may act as opsonins to facilitate phagocytosis. In our study, we controlled for this potentially confounding factor of differing levels of passive Ig levels by providing both preterm and near-term pigs the same minimal dose of passive immunity via the same volume of maternal plasma per kg body weight during the first 48 h, without any provision of their mother’s colostrum. Following this postnatal passive immunization, preterm pigs achieved a plasma IgG level of ~4 g/L, which is similar to the level in preterm infants delivered at less than 30 weeks of gestation (3–4 g/L)^32^.

During the first 4 weeks of life, preterm pigs suffered more from diarrhea and poor weight gain than piglets delivered close to term, possibly reflecting their immature digestive capacity but potentially also metabolic and immunological deficits^22^,^33^. Regardless, there were no clinical signs of NEC (e.g. abdominal distention, vomiting, bloody diarrhea) during the study period, reflecting the relatively NEC-protective diet of intact raw bovine milk used in this study, combined with some antibiotic treatments. In healthy preterm infants, the I/T ratio is quite variable but usually less than 0.22 and plasma CRP was less than 10 mg/L^34^. In our study, the I/T ratio was low during the first two weeks and was 0.24–0.27 in weeks 3–4. Plasma CRP levels were <10 mg/L in most of the pigs, and thus lower than in septic patients (>10 mg/mL^35^) and in inflamed pigs (>15 mg/mL^36^). In addition, we did not observe thrombocytopenia, a clinical sign that is often associated with NEC and sepsis^37^,^38^. These results, together with the lack of clinical signs of respiratory distress or organ failure at the end of the study, indicate that the preterm pigs in this study did not suffer from severe infections or septic conditions during the first four weeks of life. Consequently, the observed age-related changes in immune parameters likely reflect a normal, rather than a disease-stimulated, physiological maturation of the systemic immune system in preterm neonates.

Similar to newborn preterm infants^8^,^13^,^15^,^39^, newborn preterm pigs showed impaired TLR-mediated cytokine production, together with poor neutrophil phagocytic rate and NET response in cord blood. Specifically for cytokines, circulating IL-6 and IL-1 are important to induce hepatocytes to release acute phase proteins such as CRP, soluble CD14, lipopolysaccharide-binding protein and mannose-binding lectin that help to inhibit pathogen actions and detoxify microbial toxins^13^. Further, TNF-α and other cytokines may play an important role in supporting neutrophil phagocytic functions and guiding the adaptive immune response by polarizing naïve T cells into Th1, Th2, Treg or Th17^13^. Impaired cytokine production following TLR agonist challenge in preterm pigs at birth is likely due to defective expression of leukocyte receptors, such as CD14, TLRs^12^, or of second-messenger signaling intermediates, such as IRAK-4 or MyD88 proteins^8^. Interestingly, previous mechanistic studies revealed that inhibition of the intermediate IRAK-4 is derived from the action of microRNAs, such as miR146, which is highly expressed in cord blood monocytes, relative to adult monocytes^40^. On the other hand, the poor phagocytic function of neutrophils in preterm pigs at birth can partly be explained by low concentrations of opsonins in plasma, such as Igs and CRP. Further, preterm neonates also show deficient levels of blood neutrophil lactoferrin and bactericidal/permeability-increasing protein^41^,^42^. This may play a role for the impaired capacity of neutrophils to release NETs and kill bacteria, as now shown in preterm pigs, similar to recent results from preterm infants^16^.

In preterm pigs, the TLR-mediated cytokine production and plasma CRP levels increased in weeks 2–3, concomitantly with increases in leukocyte and neutrophil counts, NK cell and CD16^+^ monocyte proportions, phagocytosis and NET responses. Collectively, the data suggest that if the preterm neonate survives the first critical week, the development of more mature immune defense mechanisms in the following weeks may help to support the subsequent infection resistance. Further studies are required to investigate if the trajectory of these immunological developmental events can be modulated by nutritional, antimicrobial and medical interventions to help prevent inadequate or adverse responses to systemic bacteria.

We used elevated plasma cfDNA levels following *in vitro* stimulation, as an indicator of NET formation from neutrophils^43^, and neither preterm nor near-term cord blood had any NET response following PMA or LPS challenge. This is consistent with the observed negligible NET formation by preterm and term infant cord blood neutrophils following stimulation with PMA, LPS or platelet-activating factor^16^. When using *C. albicans* fungal challenge, term infant cord blood neutrophils exerted potent NET formation^44^, reflecting that the NET-forming capacity is partly challenge-specific. Preterm pigs demonstrated increased PMA-induced NET formation of blood neutrophils in weeks 2–3, but lower levels of LPS-induced NET formation than that found in near-term pigs. A similar trend was observed with elevated, but lower neutrophil phagocytic rates of Gram-positive bacteria in preterm versus near-term pigs during weeks 2–3. This indicates a delayed immune development in preterm pigs despite the maturation occurring after the first week. Furthermore, the dramatic increase in TLR2-mediated IL-6 and TNF-α response after the first week in preterm pigs relative to near-term pigs suggests a compensatory immune mechanism induced by the environmental challenges after preterm birth. This may facilitate additional antimicrobial mechanisms such as TNF-α-induced enhanced phagocytosis or IL-6-induced complement pathways^13^ to compensate for other delayed functions (e.g. impaired NET response).

Our work has provided new knowledge about the distinct development of the systemic immune responses in preterm neonates. The specific developmental trajectory is likely to result from both the shortened gestational age at birth and the postnatal consequences of preterm birth such as environmental exposure combined with a degree of premature organ dysfunctions. This model can be used to study interventions that may improve systemic immunity in early life of preterm neonates, including treatments to increase circulating immunoglobulin levels (passive immunization), anti-inflammatory and antimicrobial interventions, and immunomodulatory milk diets.

## Methods

### Animal experimental procedures and blood sampling

The animal procedure was approved by the Danish National Committee of Animal Experimentation. All methods in the study were performed in accordance with the approved guidelines and regulations. Preterm pigs (Large White × Danish Landrace × Duroc) were delivered by caesarean section at day 106 (n = 92, 49 male and 43 female pigs, from 5 sows, 90% of gestation, term age is 117–118 days). Caesarean-delivered near-term pigs (n = 21, 8 male and 13 female pigs, from 1 sow, day 116) and term pigs (n = 4 from 1 sow, day 118) were used for comparison. No antenatal corticosteroids were used. After delivery, piglets were transferred into individual incubators with oxygen supply (0.5–2 L/min) for the first 24 h. Preterm and near-term pigs were fed parenteral nutrition during the first week (3 days for near-term pigs, 3–7 days for preterm pigs, 48–144 mL/kg/day) via umbilical catheter, and enteral nutrition with raw bovine milk (16–200 ml/kg/day every 3 h) until 3–4 weeks (19–23 days) of age, when pigs were euthanized. Parenteral nutrition in preterm pigs was maintained for 3 days in the first two litters but was extended to 7 days in the last three litters for extra fluid provision to compensate for some degree of diarrhea and dehydration during the first week of life. Raw bovine milk, with retained bioactive components, was used as the enteral diet to avoid severe gastrointestinal complications such as NEC. Similar to preterm infants, the preterm pigs were initially fed minimal enteral nutrition with a gradual increase in feeding volume from 16 ml/kg/day (day 1) to 128 ml/kg/day (day 7), before switching to total enteral nutrition from day 8 and onwards. When there were signs of feeding intolerance, reduced boluses were given to avoid further complications. During the first 48 h after delivery, pigs were provided passive systemic immunization with maternal plasma (16–25 ml/kg). Fecal characteristics were scored twice daily: 0, no stool; 1, meconium or firm feces; 2, pasty feces; 3, diarrhea with droplets of watery feces; 4, moderate diarrhea and 5, severe diarrhea. When there were signs of dehydration and severe diarrhea, pigs were treated, for 3 consecutive days, with enteral antibiotics comprising 2.5 mg/kg gentamicin twice daily (Gentocin Vet, ScanVet, Fredensborg, Demark) and 30 mg/kg ampicillin twice daily (Norobrittin Vet, ScanVet). During the study, all preterm and near-term pigs received antibiotics during days 5–12 due to frequent diarrhea and dehydration. Details of pig housing, catheterization, ingredients of parenteral nutrition were previously described^26^. We studied the ontogeny of the innate immunity in a clinically-relevant condition for preterm infants where passive immunity is compromised, but gradual introduction of a supportive milk diet and use of antibiotics would lower the risks of NEC, infections and sepsis.

Blood samples were collected from individual pigs (cord blood at birth and jugular venous blood during weeks 2–4, on days 10–11, 19 and 23), and from their mother sows immediately after delivery. The number of animals for each blood assay during the study varied depending on the successful rate of blood sampling via the jugular vein and the available blood volume calculated based on body weight. In some experiments, sow blood and a pooled blood sample from all preterm pigs of the litter were centrifuged (2000 × g, 10 min) for plasma collection. Thereafter, plasma from preterm cord blood was mixed with cells from sow blood (after plasma removal), to form an artificial sample denoted ‘sow cells with preterm plasma (S cells/P plasma)’. Similarly, sow plasma was mixed with cells from preterm cord blood to form a sample of ‘preterm cells with sow plasma (P cells/S plasma)’. For most endpoints, the measured blood values were similar for weeks 2–4, and for simplicity, we presented the pooled values across this period.

### Blood leukocyte counts, leukocyte phenotyping and plasma mediators

Blood samples from preterm, near-term, term and adult pigs (four mother pigs after delivery and seven healthy adult pigs without pregnancy) were collected for blood leukocyte counting by an automatic cell counter (Advia 2120i Hematology System, Siemens, Germany). Plasma samples from preterm pigs over the four weeks after birth were analyzed for CRP and IgG levels by ELISA as previously described^45^,^46^.

Preterm blood aliquots of 100 μl were used for cell phenotyping. Erythrocytes were lyzed (1 × BD FACS Lysing solution, BD Biosciences), then leukocytes were washed, blocked by porcine serum and stained for either one of two antibody combinations. Combination 1 included PE-labelled mouse anti-pig CD172a (BD Biosciences), FITC-labelled mouse anti-pig 2B2 and mouse anti-pig 6D10 conjugated with PerCP-Cy5.5 (AbD Serotec, Kidlington, UK and Santa Cruz Biotechnology, CA, USA). Combination 2 included PE-labelled mouse anti-pig CD172a and FITC-labelled mouse anti-pig CD16 (AbD Serotec). FITC-labelled mouse IgG1 (AbD Serotec) and PE-labelled mouse IgG2b (eBioscience, San Diego, CA, USA) were used as isotype controls. Stained cells were fixed in 4% formalin and analyzed by flow cytometry by FACS Canto II flow cytometer (BD Biosciences, USA). Staining profiles were analyzed by FlowJo (Tree Star Inc., Oregon, USA). Gating strategy was performed as previously described^47^,^48^ and as demonstrated in Fig. 1. In brief, gates for major subsets were set based on forward scatter (FSC), side scatter (SSC) and CD172a^47^: granulocytes with FSC^high^SSC^high^CD172a^med^, monocytes with FSC^high^SSC^med^CD172a^high^, and lymphocytes with FSC^med^SSC^low^CD172a^−^. Further, immature neutrophils (6D10^+^2B2^−^ neutrophils) and mature neutrophils (6D10^+^2B2^+^ neutrophils) were gated^48^. NK cells were identified as CD16^+^ lymphocytes.

### TLR and NOD receptors-mediated cytokine production in whole blood

To characterize the ability to regulate systemic inflammatory response in newborn preterm pigs, a pooled cord blood sample from all preterm pigs and a venous blood sample of the corresponding sow after delivery were collected for each of three separate litters (n = 3). Whole blood (200 μl) was stimulated with 10 different TLR and NOD agonists (Invivogen, San Diego, CA, USA) at different agonist concentrations at 37 °C, 5% CO2 for 5 h: FSL-1 (TLR2/6 agonist, 10^−4^–10^2^ ng/mL), Pam_3_CSK_4_ (TLR1/2 agonist, 10^−4^–10^2^ ng/mL), heat-killed *Listeria monocytogenes* (HKLM, TLR2 agonist, 10^2^–10^8^ cells/mL), Poly I:C (TLR3 agonist, 10^−2^–10^4^ ng/mL), LPS (TLR4 agonist, 10^−3^–10^3^ ng/mL), Flagellin (TLR5 agonist, 10^−4^–10^2^ ng/mL), Imiquimod (TLR7 agonist, 10^−2^–10^4^ ng/mL), ODN:2216 (TLR9 agonist, 10^−2^–10^4^ ng/mL), C12-IE-DAP peptide (NOD1 agonist, 10^−2^–10^4^ ng/mL), and MDP peptide (NOD2 agonist, 10^−2^–10^4^ ng/mL). Thereafter, blood was diluted 5-fold with RPMI 1640 medium (Life Technologies, Nærum, Denmark), centrifuged at 2000 × g, 4 °C for 10 min. Supernatants were collected for analysis of IL-6 and TNF-α by ELISA (R&D Systems, Abingdon, UK).

Experiments with TLR2 agonist were also performed on venous blood collected during weeks 2–3. Due to the limited volume of blood available from preterm pigs, blood was randomly pooled for each 3–4 pigs to obtain 6 pools per litter. Blood was stimulated with TLR2 agonist (10^7^ HKLM cells/mL) for IL-6 and TNF-α analysis as mentioned above.

### Neutrophil phagocytic rate in the blood

Blood samples from preterm and near-term pigs (at birth and during weeks 2–3), from sow, and samples of P cells/S plasma and S cells/P plasma were tested for neutrophil phagocytosis against both Gram-negative and Gram-positive bacteria using pHrodo Red *E. coli* (560/585 nm) or pHrodo Green *S. aureus* (509/533 nm) BioParticles Phagocytosis Kits for Flow cytometry (Life Technologies) following the manufacturer’s instruction. pHrodo is non-fluorescent at neutral pH but it turns red or green (for pHrodo Red or Green particles) in acidic intracellular conditions. Briefly, blood was stimulated with pHrodo Red-conjugated *E. coli* or pHrodo Green-conjugated *S. aureus* at approximately 10:1 particle-to-phagocyte, 37 °C, 5% CO_2_ for 30 min. Thereafter, erythrocytes were remove and leukocytes were washed and analyzed by FACS Canto II flow cytometer. Samples with no treatment and with treatments incubated at 4 °C were used as controls. Neutrophil population was gated based on FSC vs. SSC plot with FSC^high^SSC^high^. The proportion of pHrodo^+^ neutrophils (%) in the neutrophil population indicates proportion of neutrophils exerting phagocytosis (neutrophil phagocytic rate).

### Ability to release circulating neutrophil extracellular traps (NETs)

Whole blood samples (200 μl) obtained from all preterm and near-term pigs at birth and during weeks 2–3 were stimulated with 100 ng/mL PMA or 0.1–1 μg/mL LPS (both from Sigma Aldrich, Denmark) for 3 h at 37 °C 5% CO_2_. Plasma was collected (2,000 × g, 4 °C,10 min) for analysis of cell-free DNA (cfDNA), a surrogate marker of NETs, using Quant-iT PicoGreen dsDNA kit (Life Technologies). NET response is quantified as a relative fold-change of plasma cfDNA concentrations in samples after stimulation with that in control samples.

The ability to produce circulating NETs *in vivo* was also evaluated via plasma cfDNA levels in newborn preterm pigs 6–12 h after systemic injection of *S. epidermidis* (5 × 10^9^ CFU/kg) immediately after birth (six pigs with *S. epidermidis* injection, six pigs with control saline injection).

### Statistics

All statistical analyses were performed using JMP version 10.0 (SAS Institute, Cary, NC, USA). For comparison of TLR/NOD-mediated cytokine production between preterm vs. sow, cytokine data were fitted into a linear model with pig type (preterm or sow), litter and concentrations of agonists as fixed factors. Phagocytosis, cfDNA and blood leukocyte count parameters for comparison among preterm and near-term pigs at birth and sow were fitted in a linear model with cell type (preterm, term, sow, preterm cells/sow plasma, sow cells/preterm plasma) as a fixed factor, followed by post hoc Tukey test. Temporal parameters were fitted in a linear mixed model with postnatal age, gender and delivery time (preterm or near-term, when necessary) as fixed factors and pig code as a random factor, followed by a post hoc Tukey test or sliced F test. Values are means ± SEM. P < 0.05 is regarded as statistical significance.

## Additional Information

**How to cite this article**: Nguyen, D. N. *et al*. Delayed development of systemic immunity in preterm pigs as a model for preterm infants. *Sci. Rep.* **6**, 36816; doi: 10.1038/srep36816 (2016).

**Publisher’s note:** Springer Nature remains neutral with regard to jurisdictional claims in published maps and institutional affiliations.

## Supplementary Material

Supplementary Information

## References

[b1] Strunk T. . Infection-induced inflammation and cerebral injury in preterm infants. Lancet Infect Dis 14, 751–762 (2014). 24877996 10.1016/S1473-3099(14)70710-8 PMC4125363

[b2] Hackam D. J., Afrazi A., Good M. & Sodhi C. P. Innate immune signaling in the pathogenesis of necrotizing enterocolitis. Clin Dev Immunol 2013, 475415 (2013). 23762089 10.1155/2013/475415 PMC3677005

[b3] Neu J. & Walker W. A. Necrotizing enterocolitis. N. Engl. J. Med. 364, 255–264 (2011). 21247316 10.1056/NEJMra1005408 PMC3628622

[b4] Azizia M., Lloyd J., Allen M., Klein N. & Peebles D. Immune status in very preterm neonates. Pediatrics 129, e967–e974 (2012). 22451711 10.1542/peds.2011-1579

[b5] Lim J. C., Golden J. M. & Ford H. R. Pathogenesis of neonatal necrotizing enterocolitis. Pediatr Surg Int 31, 509–518 (2015). 25854935 10.1007/s00383-015-3697-9

[b6] Nussbaum C. & Sperandio M. Innate immune cell recruitment in the fetus and neonate. J Reprod Immunol 90, 74–81 (2011). 21641657 10.1016/j.jri.2011.01.022

[b7] Cortese F. . Early and Late Infections in Newborns: Where Do We Stand? A Review. Pediatr Neonatol, doi: 10.1016/j.pedneo.2015.09.007 (2015). 26750406

[b8] Cuenca A. G., Wynn J. L., Moldawer L. L. & Levy O. Role of innate immunity in neonatal infection. Am J Perinatol 30, 105–112 (2013). 23297181 10.1055/s-0032-1333412 PMC3959733

[b9] Nguyen D. N. . Oral antibiotics increase blood neutrophil maturation and reduce bacteremia and necrotizing enterocolitis in the immediate postnatal period of preterm pigs. Innate Immun 22, 51–62 (2016). 26561386 10.1177/1753425915615195

[b10] Walker M. Formula Supplementation of the breastfed infant: assault on the gut microbiome. Clin Lact 5, 128–132 (2014).

[b11] Ng P. C. . Host-response biomarkers for diagnosis of late-onset septicemia and necrotizing enterocolitis in preterm infants. J Clin Invest 120, 2989–3000 (2010). 20592468 10.1172/JCI40196 PMC2912182

[b12] Tissières P. . Innate immune deficiency of extremely premature neonates can be reversed by interferon-γ. PLoS ONE 7, e32863 (2012). 22427899 10.1371/journal.pone.0032863 PMC3299693

[b13] Levy O. Innate immunity of the newborn: basic mechanisms and clinical correlates. Nat Rev Immunol 7, 379–390 (2007). 17457344 10.1038/nri2075

[b14] Battersby A. J., Khara J., Wright V. J., Levy O. & Kampmann B. Antimicrobial proteins and peptides in early life: ontogeny and translational opportunities. Front Immunol 7, 309 (2016). 27588020 10.3389/fimmu.2016.00309 PMC4989132

[b15] Prosser A. . Phagocytosis of neonatal pathogens by peripheral blood neutrophils and monocytes from newborn preterm and term infants. Pediatr Res 74, 503–510 (2013). 23999070 10.1038/pr.2013.145

[b16] Yost C. C. . Impaired neutrophil extracellular trap (NET) formation: a novel innate immune deficiency of human neonates. Blood 113, 6419–6427 (2009). 19221037 10.1182/blood-2008-07-171629 PMC2710935

[b17] Sangild P. T. . Invited review: the preterm pig as a model in pediatric gastroenterology. J Anim Sci 91, 4713–4729 (2013). 23942716 10.2527/jas.2013-6359 PMC3984402

[b18] Jensen M. L. . Similar efficacy of human banked milk and bovine colostrum to decrease incidence of necrotizing enterocolitis in preterm piglets. Am J Physiol Regul Integr Comp Physiol 305, R4–R12 (2013). 23657639 10.1152/ajpregu.00094.2013

[b19] Brinkmann V. . Neutrophil extracellular traps kill bacteria. Science 303, 1532–1535 (2004). 15001782 10.1126/science.1092385

[b20] József L., Khreiss T., Kebir D. E. & Filep J. G. Activation of TLR-9 Induces IL-8 Secretion through Peroxynitrite Signaling in Human Neutrophils. J Immunol 176, 1195–1202 (2006). 16394009 10.4049/jimmunol.176.2.1195

[b21] Yee W. H. . Incidence and timing of presentation of necrotizing enterocolitis in preterm infants. Pediatrics 129, e298–e304 (2012). 22271701 10.1542/peds.2011-2022

[b22] Andersen A. D. . Delayed growth, motor function and learning in preterm pigs during early postnatal life. Am J Physiol Regul Integr Comp Physiol 310, 481–492 (2016). 10.1152/ajpregu.00349.2015 26764054

[b23] Cerwenka A. & Lanier L. L. Natural killer cells, viruses and cancer. Nat Rev Immunol 1, 41–49 (2001). 11905813 10.1038/35095564

[b24] Connor R. I. . Fc receptors for IgG (Fc gamma Rs) on human monocytes and macrophages are not infectivity receptors for human immunodeficiency virus type 1 (HIV-1): studies using bispecific antibodies to target HIV-1 to various myeloid cell surface molecules, including the Fc gamma R. Proc Natl Acad Sci USA 88, 9593–9597 (1991). 1835086 10.1073/pnas.88.21.9593 PMC52764

[b25] Sangild P. T. . Diet- and colonization-dependent intestinal dysfunction predisposes to necrotizing enterocolitis in preterm pigs. Gastroenterology 130, 1776–1792 (2006). 16697741 10.1053/j.gastro.2006.02.026

[b26] Jensen M. L. . Antibiotics modulate intestinal immunity and prevent necrotizing enterocolitis in preterm neonatal piglets. Am J Physiol Gastrointest Liver Physiol 306, G59–G71 (2014). 24157972 10.1152/ajpgi.00213.2013 PMC4073901

[b27] Birck M. M. . Enteral but not parenteral antibiotics enhance gut function and prevent necrotizing enterocolitis in formula-fed newborn preterm pigs. Am J Physiol Gastrointest Liver Physiol 310, 323–333 (2015). 10.1152/ajpgi.00392.2015 26680737

[b28] van den Berg J. P., Westerbeek E. a. M., van der Klis F. R. M., Berbers G. a. M. & van Elburg R. M. Transplacental transport of IgG antibodies to preterm infants: a review of the literature. Early Hum Dev 87, 67–72 (2011). 21123010 10.1016/j.earlhumdev.2010.11.003

[b29] Sangild P. T., Trahair J. F., Loftager M. K. & Fowden A. L. Intestinal macromolecule absorption in the fetal pig after infusion of colostrum in utero. Pediatr Res 45, 595–602 (1999). 10203154 10.1203/00006450-199904010-00021

[b30] Kim Y. B. Developmental immunity in the piglet. Birth Defects Orig. Artic. Ser. 11, 549–557 (1975). 1096995

[b31] Sangild P. T. . Preterm birth affects the intestinal response to parenteral and enteral nutrition in newborn pigs. J Nutr 132, 3786–3794 (2002). 12492087 10.1093/jn/132.9.2673

[b32] Hyvarinen M., Zeltzer P., Oh W. & Stiehm E. R. Influence of gestational age on serum levels of alpha-1 fetoprotein, IgG globulin, and albumin in newborn infants. J Pediatr 82, 430–437 (1973). 4121530 10.1016/s0022-3476(73)80116-7

[b33] Hansen C. F. . Rapid gut growth but persistent delay in digestive function in the postnatal period of preterm pigs. Am. J. Physiol. Gastrointest. Liver Physiol. 310, 550–560 (2016). 10.1152/ajpgi.00221.2015 PMC4836131 26822913

[b34] Polin R. A. . Management of neonates with suspected or proven early-onset bacterial sepsis. Pediatrics 129, 1006–1015 (2012). 22547779 10.1542/peds.2012-0541

[b35] Póvoa P. C-reactive protein: a valuable marker of sepsis. Intensive Care Med 28, 235–243 (2002). 11904651 10.1007/s00134-002-1209-6

[b36] Bürger W., Fennert E. M., Pohle M. & Wesemeier H. C-reactive protein–a characteristic feature of health control in swine. Zentralbl Veterinarmed A 39, 635–638 (1992). 1455931 10.1111/j.1439-0442.1992.tb00227.x

[b37] Maheshwari A. Immunologic and hematological abnormalities in necrotizing enterocolitis. Clin Perinatol 42, 567–585 (2015). 26250918 10.1016/j.clp.2015.04.014 PMC4530495

[b38] Manzoni P. Hematologic aspects of early and late-onset sepsis in preterm infants. Clin Perinatol 42, 587–595 (2015). 26250919 10.1016/j.clp.2015.04.012

[b39] Sadeghi K. . Immaturity of infection control in preterm and term newborns is associated with impaired Toll-Like receptor signaling. J Infect Dis 195, 296–302 (2007). 17191175 10.1086/509892

[b40] Lederhuber H. . MicroRNA-146: tiny player in neonatal innate immunity? Neonatology 99, 51–56 (2011). 20616571 10.1159/000301938

[b41] Ambruso D. R., Bentwood B., Henson P. M. & Johnston R. B. Oxidative metabolism of cord blood neutrophils: relationship to content and degranulation of cytoplasmic granules. Pediatr Res 18, 1148–1153 (1984). 6096799 10.1203/00006450-198411000-00019

[b42] Levy O. . Impaired innate immunity in the newborn: newborn neutrophils are deficient in bactericidal/permeability-increasing protein. Pediatrics 104, 1327–1333 (1999). 10585984 10.1542/peds.104.6.1327

[b43] Liaw P. C., Ito T., Iba T., Thachil J. & Zeerleder S. DAMP and DIC: The role of extracellular DNA and DNA-binding proteins in the pathogenesis of DIC. Blood Rev 30, 257–261 (2015). 26776504 10.1016/j.blre.2015.12.004

[b44] Byrd A. S. . NETosis in Neonates: Evidence of a reactive oxygen species-independent pathway in response to fungal challenge. J Infect Dis 213, 634–639 (2016). 26333942 10.1093/infdis/jiv435 PMC4721906

[b45] Heegaard P. M. H., Pedersen H. G., Jensen A. L. & Boas U. A robust quantitative solid phase immunoassay for the acute phase protein C-reactive protein (CRP) based on cytidine 5′-diphosphocholine coupled dendrimers. J Immunol Methods 343, 112–118 (2009). 19236874 10.1016/j.jim.2009.02.002

[b46] Henryon M., Heegaard P. M. H., Nielsen J., Berg P. & Juul-Madsen H. R. Immunological traits have the potential to improve selection of pigs for resistance to clinical and subclinical disease. Anim Sci 82, 597–606 (2006).

[b47] Zelnickova P., Faldyna M., Stepanova H., Ondracek J. & Kovaru F. Intracellular cytokine detection by flow cytometry in pigs: Fixation, permeabilization and cell surface staining. J Immunol Methods 327, 18–29 (2007). 17720184 10.1016/j.jim.2007.07.006

[b48] Pérez C. . Phenotypic and functional characterization of porcine granulocyte developmental stages using two new markers. Dev Comp Immunol 31, 296–306 (2007). 16919332 10.1016/j.dci.2006.06.002

