# Supplementary material for: Delayed development of systemic immunity in preterm pigs as a model for preterm infants
Source: Sci Rep. 2016 Nov 10;6:36816. doi: 10.1038/srep36816 (PMC5103294; doi:10.1038/srep36816)
Supplement: Supplementary Information [file srep36816-s1.pdf]

Supplementary information

Delayed development of systemic immunity in preterm pigs as a model of preterm infants

Duc Ninh Nguyen[1], Pingping Jiang[1], Hanne Frøkiær[2], Peter M.H. Heegaard[3], Thomas Thymann[1], Per T. Sangild[1,4*]

[1]*Section of Comparative Pediatrics and Nutrition, Department of Veterinary Clinical and Animal Sciences, University of Copenhagen, Denmark*

[2] *Department of Veterinary Disease Biology, University of Copenhagen, Denmark*

[3] *National Veterinary Institute, Technical University of Denmark, Frederiksberg, Denmark*

[4] *Department of Pediatrics and Adolescent Medicine, Rigshospitalet, Copenhagen, Denmark*

Corresponding author: Per T. Sangild, Section of Comparative Pediatrics and Nutrition, Department of Veterinary Clinical and Animal Sciences, University of Copenhagen, Dyrlægevej 68, DK-1870 Frederiksberg C, Denmark. Tel: +45 35 33 26 98, Fax: +45 35 33 24 83, Email: pts@sund.ku.dk

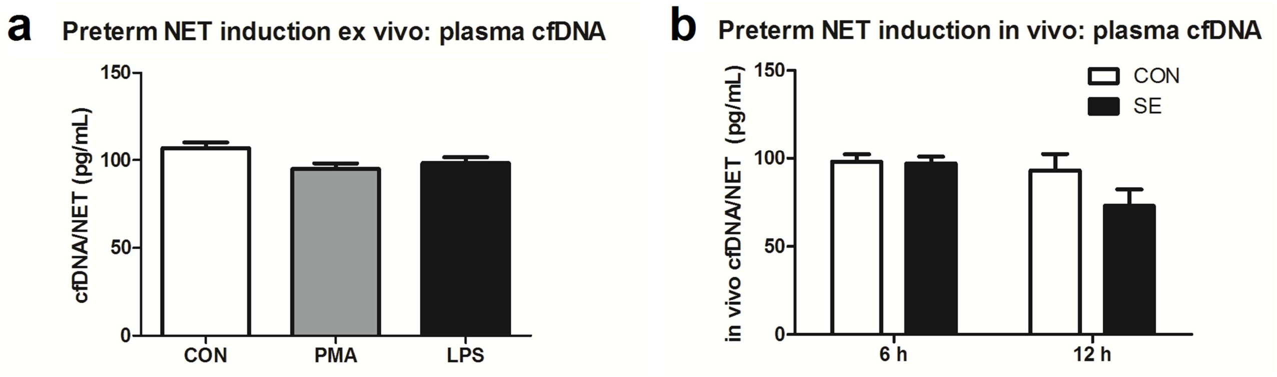

Supplementary Figure S1: **Impairment of *ex vivo* and *in vivo* NET production in newborn preterm pigs.** a: Plasma cfDNA levels following *ex vivo* stimulation of whole blood with 100 ng/mL PMA or 1 µg/mL LPS for 3 h (n = 61). b: Plasma cfDNA levels from pigs following infusion of systemic saline or *S.epidermidis* (5 x 10^9 cells/kg) for 6-12 h (n = 6). SE: *S.epidermidis*
